# From theory to practice – assessing translation of physical fitness research in the emergency department through machine learning and natural language processing

**DOI:** 10.1017/cts.2025.10051

**Published:** 2025-05-21

**Authors:** Kristin Morrow, Debajyoti Datta, Lindsey Spiegelman, Roy Almog, Kai Zheng, Don Brown, Dan Michael Cooper

**Affiliations:** 1 School of Engineering, University of Virginia, VA, USA; 2 Department of Emergency Medicine, University of California, Irvine, CA, USA; 3 Department of Informatics, University of California, Irvine, CA, USA; 4 School of Data Science, University of Virginia, VA, USA; 5 UCI Institute for Precision Health, University of California, Irvine, CA, USA

**Keywords:** Natural language processing, text analysis, semantic similarity, translation research, physical fitness

## Abstract

**Background:**

A critical challenge for biomedical investigators is the delay between research and its adoption, yet there are few tools that use bibliometrics and artificial intelligence to address this translational gap. We built a tool to quantify translation of clinical investigation using novel approaches to identify themes in published clinical trials from PubMed and their appearance in the natural language elements of the electronic health record (EHR).

**Methods:**

As a use case, we selected the translation of known health effects of exercise for heart disease, as found in published clinical trials, with the appearance of these themes in the EHR of heart disease patients seen in an emergency department (ED). We present a self-supervised framework that quantifies semantic similarity of themes within the EHR.

**Results:**

We found that 12.7% of the clinical trial abstracts dataset recommended aerobic exercise or strength training. Of the ED treatment plans, 19.2% related to heart disease. Of these, the treatment plans that included heart disease identified aerobic exercise or strength training only 0.34% of the time. Treatment plans from the overall ED dataset mentioned aerobic exercise or strength training less than 5% of the time.

**Conclusions:**

Having access to publicly available clinical research and associated EHR data, including clinician notes and after-visit summaries, provided a unique opportunity to assess the adoption of clinical research in medical practice. This approach can be used for a variety of clinical conditions, and if assessed over time could measure implementation effectiveness of quality improvement strategies and clinical guidelines.

## Introduction

The primary goal of this research was to develop an innovative and reproducible framework that leverages informatics and natural language processing (NLP) techniques to quantify the potential translation of research into clinical practice. To achieve this, we used topic modeling, clustering algorithms, text summarization, and similarity search into a cohesive framework designed to track the propagation of a research signal throughout the literature. Recognizing the challenges that still exist in searching biomedical publication databases [1] and in exploring natural language in electronic health records (EHR) [2], we implemented a novel approach for identifying disease-specific themes in PubMed research articles and for searching for these themes in the EHR. As a feasibility use case, we applied this approach to quantify and compare the appearance of themes related to exercise and physical fitness found in PubMed publications reporting clinical trials with similar themes in the free-text clinical narratives of patients seen in an emergency department (ED) of an academic health center and diagnosed with heart disease.

Translating relevant biomedical discoveries into clinical practice is an unacceptably arduous and lengthy process [3]. Proctor et al. recently highlighted the need to develop novel metrics and approaches that quantify the speed and success of clinical implementation of new discovery. Their Framework to Assess Speed of Translation of biomedical innovation is at the very early stages of development and the authors noted, ”we have lacked careful, explicit study of determinants of implementation speed, as well as an agenda to build a more robust knowledge base that reduces the time from discovery to widespread use.”[4] In this study, we developed an AI-based informatics approach leveraging self-supervised learning to improve query retrieval without training on PubMed and EHR data. Self-supervised learning is a machine learning technique increasingly employed in the analysis of EHR data [5] where the model learns to perform tasks without labeled data. By generating its own labels from the data, the model can process and understand unseen data, making the query retrieval process more efficient and streamlined.

Our focus on the physical fitness use case was driven, in part, by an unfortunate development associated with the COVID-19 pandemic and the related shutdowns. There has been an increase in obesity and poor cardiorespiratory fitness (CRF) across the lifespan [6,7] Given that exercise and physical activity play an important role in the treatment and prevention of cardiovascular diseases [8,9], addressing these trends is essential. While ED visits decreased during the height of the pandemic, the rebound in ED visits in states like California has exceeded capacity [10]. The cost of poor physical fitness and associated obesity is tremendous, and ED physicians and nurses providing brief lifestyle counseling to appropriate patients could be effective. Moreover, approaches to the exercise prescription at the interface of the patient provider are improving [11]. Our approach offers a means to assess the impact of implementing the exercise prescription even in the context of a busy ED.

Emergency medicine physicians are at the frontline of clinical care, attend to a bewildering array of health issues, and strive to implement the most up-to-date, evidence-based therapeutic approaches. Logistical challenges, heavy workloads, and the high volume of patients in the ED often limit the attention given to complex lifestyle-related diseases, such as obesity, and physical fitness-related conditions, which disproportionately affect many of the typical patients seen in a busy ED [12]. Comprehensive discussions with patients focused on changing lifestyle patterns of exercise or diet in a busy ED are certainly challenging [13,14] However, there are emerging data that exercise prescriptions [15] and brief motivational interviewing can be effective in the ED [16,17] Given the nature of ED encounters, we hypothesized modest thematic similarity between physical activity or exercise between published clinical trials and free-text records from the EHR.

### Data: PubMed

We searched PubMed journal abstracts in the “clinical trial” category to capture publications focused on physical fitness and disease, health conditions, or medical decision-making using the following search query: (((“physical fitness”) OR (“physical activity”) OR (fitness) OR (exercise)) AND (health) AND (((diagnosis) OR (diagnose))) from 2002 to 2022. The query returned around 109,000 abstracts. We filtered the dataset to include only clinical trial article types that contained research results. After filtering, the dataset included 15,639 abstracts.

### Data: EHR

We acquired EHR data from the ED of University of California, Irvine (UCI) Health. The UCI Health ED is the only level 1 trauma center in the area, as well as a Stroke and STEMI receiving center. A level 1 Trauma Center is a comprehensive regional resource that is a tertiary care facility central to the trauma system and capable of providing total care for every aspect of injury – from prevention to rehabilitation. The ED includes a diverse range of visit types, providing a comprehensive representation of patient experiences. According to our coauthors, Spiegelman and Almog, an estimated 50% to 60% of these visits are nonacute and can be classified as non-emergencies. The EHR system at UCI Health is Epic (Epic Systems, Verona, WI). For this research, honest brokers retrieved relevant patient data from Epic and provided the data to the researchers in a de-identified format. They included all types of free-text documents associated with ED visits, including discharge summaries, between February 2019 and February 2022, focusing on those containing exercise-related keywords. Given the critical role of CRF in health outcomes and the wide variation in patient background and acuity of presentation, this study has chosen to focus on CRF. This query resulted in 24,198 unique patient records. To hone in on relevant results, we identified records that contained phrasing around treatment plans, therapy recommendations, or patient instructions, resulting in an EHR dataset of 13,720 patient records.

## Methods

**Figure 1.**
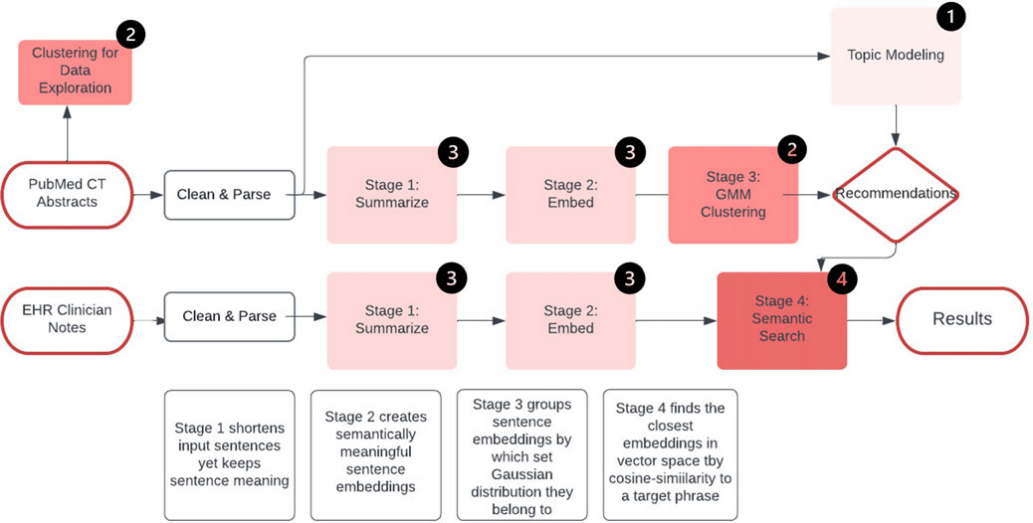
Data pipeline. **Legend:** Clinical trials (CT), Gaussian mixture model (GMM).

This study was deemed nonhuman subject research by the UCI IRB and patient consent was not required. Figure 1 illustrates the entire methodology of our approach.

### Topic modeling on disease types

**Figure 2.**
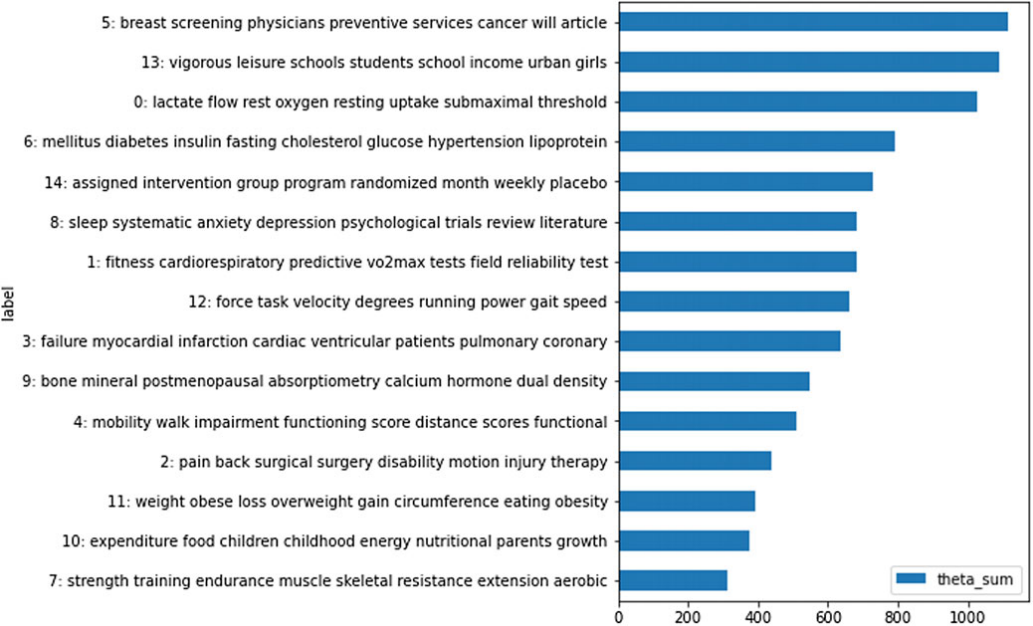
Top 15 Topics in PubMed clinical trial data: the *Y* axis shows the words that make up each topic, while the *X* axis represents how prevalent that topic is to the overall corpus.

To explore themes appearing in the PubMed clinical trial abstracts, we used scikit-learn’s latent Dirichlet allocation (LDA) topic modeling to identify underlying topics. LDA is an unsupervised machine learning algorithm that uses a probabilistic model to build on Dirichlet distributions [18]. LDA allows for multiple topics in a document and each topic is considered a probability distribution over the set of words [18]. We explored the top 15 topics by volume, represented by the ten most occurring words for each topic as shown in Figure 2. Three disease types appeared in different topics: heart disease in topic 3, diabetes in topic 6, and mental health in topic 8. For this study, we selected heart disease from topic 3 to use throughout PubMed and EHR analysis.

### Clustering

Unlike topic modeling, clustering splits documents into a number of groups, or clusters, based on a similarity score. We tested multiple clustering algorithms to see which worked best with our datasets and then explored the clusters of the best-performing technique. We chose three clustering algorithms: K-Means [19], Gaussian mixture model (GMM) [20], and spectral clustering [21]. We evaluated all models for clusters ranging from three to ten and ranked them by highest Silhouette score and lowest Davies Bouldin score. We chose the Silhouette and Davies Bouldin scores as our definition of cluster quality. Silhouette score measures cluster quality by how well the data fits within each cluster; higher-valued scores mean the clusters are strong and the points within that cluster are the most similar to the other points within that cluster. The Davies Bouldin score measures the average similarity between one cluster and the closest neighboring cluster; lower values mean stronger clusters because it means the clusters are more distinct from each other. K-Means and GMM performed best with nine clusters, while spectral performed best with 7. Of the three clustering algorithms tested, the GMM with full covariance performed the best overall, with a Silhouette score of 0.066 and a Davies Bouldin score of 2.948. Full covariance allows each cluster component to take on any shape and orientation independently of other clusters.

### Sentence transformers

Sentence transformers are a form of deep learning that provide streamlined methods to compute embeddings, or dense vector representations, for text and image data [22]. These representations allow the meaning and context of the data to be captured, which further allows for the processing of large amounts of unstructured data. By representing text as vectors in high-dimensional space, a piece of text can be positioned closer to other pieces of text that have a similar meaning. We measure closeness by cosine similarity:
(1)

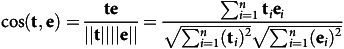




In this equation, cos(*t,e*) represents the cosine of the angle between vectors t and e, which is equal to the dot product of t and e divided by the product of their magnitudes. Vectors t and e can be represented as column vectors of their respective components. Sentence transformers make it easy to compare, cluster, and retrieve text quickly. Our research used sentence transformers for summarization, embedding, and semantic similarity searches.

### Summarization and embedding

The text summarization process creates new sentences based on input text that are shorter and more concise while still capturing the main ideas of the original text. We first fed the abstract results and EHR treatment plans into a summarization model. All the results and treatment plans were then summarized and capped at a maximum length of 25. After using sentence transformers to summarize abstracts and treatment plans, we used them to embed summarized sentences with the all-MiniLM-L6-v2 model [23]. Embedding is an NLP technique that transforms unstructured data, such as text or images, into a structured and numerical form, in this case as a high-dimensional numerical vector. The embedding process was identical for both datasets, except that duplicates (i.e., identical treatment plans duplicated between visits and/or patients) were not removed from the EHR data.

### Similarity search

We then searched for semantic similarity between themes found in the PubMed clinical trials dataset and the EHR datasets. A similarity search matches relevant pieces of data together using features or characteristics of the item of interest, in this case a target sentence. We selected target sentences around different exercise interventions recommended from PubMed clinical trials that we identified with clustering analysis. We searched for these exercise interventions in the summarized treatment plans from the EHR data. We utilized Facebook artificial intelligence (AI) Similarity Search, or FAISS, a computationally efficient vector database [24]. FAISS has a set of algorithms to search in dense vector sets of any size to compute the argmin on the data based on the target sentence or query vector [25]. From there, the distance between each returned sentence and the target sentence is measured, and the returned sentence with the smallest distance from the target is considered the most similar. For our research, we chose FAISS to measure the Euclidean distance between all points between the target sentence and the vectors in the index.

## Models

### Summarization models

We selected the T5-Base transformer model with its unified text-to-text format for summarization tasks [26]. The T5-base model passes an input sequence of tokens into embeddings, which then pass to an encoder. The encoder is made up of a self-attention layer and a small feed-forward network layer with layer normalization applied to both. Next, a residual skip connection adds each layer’s input to its output and applies dropout to the feed-forward network, the skip connection, the attention weights, and the input and output of the entire stack. The decoder is structured similarly to the encoder. However, it includes a standard attention mechanism after every self-attention layer to attend to the output of the encoder and only to other past outputs. Lastly, the final decoder block output feeds to a dense layer with a soft-max output whose weights are shared with the input embeddings [27]. Based on our qualitative review of summarized sentences, we found no serious loss of information. This is especially true for the already condensed PubMed results sections.

### Embedding models

The primary goal of an embedding model is to transform entire sentences into a structured representation while keeping their meaning. We wanted to make use of embedding models without having to do any additional training on the data. We considered three different sentence transformers for embedding to see which performed the best in a self-supervised learning approach. All models considered utilized a self-supervised, contrastive learning objective. Contrastive learning involves training a model to correctly identify which sentence, from a randomly sampled set, is paired with a given sentence in the dataset. We provide the contrastive learning equation below:
(2)

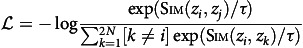




Where L is the loss function, *z*_*i*_ and *z*_*j*_ are embeddings of two augmented versions of an input, Sim (*z*_*i*_ *,z*_*j*_) is a similarity function that measures the similarity between the two embeddings, *τ* is a temperature parameter, and *N* is the number of samples [28].

The transformers analyzed were all-MiniLM-L6-v2, multi-qa-mpnet-base-dot-v1 [29], and all-mpnet-base-v2 [30]. We selected the all-MiniLM-L6-v2 model because it: 1) is derived from the all-mpnet-base-v2 model, which has the best quality rated by SBERT [31], 2) performs the highest in both performance sentence embeddings and performance semantic search, 3) is a smaller version of the all-mpnet-base-v2 model, performing 5x faster while retaining good quality, and 4) is intended for both clustering and semantic search, both used throughout our research.

Using only the pretraining of the entire MiniLM-L6-v2 model, we utilized a self-supervised learning framework that could generate meaningful results without the need to acquire additional domain-specific data or provide labeled data. This approach is particularly powerful in the healthcare domain, where acquiring and labeling medical data remains challenging.

### Clustering

To group abstract results sections with similar themes, we used GMM clustering on the word embeddings. The different clusters allowed us to explore and analyze similar recommendations in the clinical trial results that we could then look for in the EHR data. After our first round of GMM clustering, the GMM with five cluster components and diagonal covariance performed the best, with a Silhouette score of 0.036 and a Davies Bouldin score of 3.917. A diagonal covariance is when the contour axes are oriented along the coordinate axes, but other eccentricities may vary. GMM clustering with diagonal covariance performed the best on the word embeddings, whereas full covariance performed the best during data exploration on the word tokens. We generated the GMM and assigned cluster labels to each abstract’s results section so that the clusters could be grouped and explored. The cluster themes from the 5-component GMM focused primarily on the specific wording of the results sections, such as whether or not the findings were conclusive or significant or if any change was observed and not on the actual content of the results.

To address this, we performed another round of clustering to group by whether the clusters from the GMM with five components had conclusive results; this left clusters 0, 1, and 2 and a total of 10,791 clinical trial abstract results. We combined clusters 0, 1, and 2, and again GMMs were evaluated based on components ranging from three through ten with diagonal covariance. The GMM with three cluster components performed the best, with a Silhouette score of 0.039 and a Davies Bouldin score of 4.609. We explored the clusters to pull out underlying themes, and this time clusters revolved around intervention types. Cluster 0 focused on weight management techniques and dieting, cluster 1 focused on counseling, therapy, breathing techniques, and yoga, and cluster 2 focused on exercise programs and improving muscle strength.

The final round of clustering focused on heart disease specifically. Having selected heart disease through topic modeling, we filtered for it indirectly through keyword searches. Guided by the UCI Health emergency physician investigators, we compiled a list that included different naming conventions and symptoms. Clustering was performed a third time on each of the grouped abstracts by the heart disease filter. We saw similar cluster themes around different interventions such as education, diet, and exercise. We chose the intervention types for each condition based on the most occurring and most relevant interventions. Consequently, we selected “aerobics” and “strength training” for heart disease. Aerobics and strength training were highlighted when clustering abstract results sections, but also appeared in topic seven from topic modeling.

## Results

We found that 12.7% of all clinical trial abstracts mentioned aerobic exercise or strength training. Of the treatment plans we reviewed from UCI Health’s ED, 19.2% related to heart disease. Of this subset, treatment plans with heart disease only mentioned aerobic exercise or strength training 0.34% of the time. Lastly, treatment plans from the overall dataset, not filtered for heart disease specifically, from UCI Health mentioned aerobic exercise or strength training less than 5% of the time.

### PubMed recommendation table

To ensure that we included all relevant, heart disease-related PubMed clinical trial abstracts, we used the same keyword filter for heart disease as we did in the last round of clustering analysis and filtered the on the entire abstract text instead of just the summarized results sections. Broadening our search criteria to the entire PubMed abstract increased the number of clinical trial abstracts related to heart disease from 443 to 2,629. We then performed another round of keyword searches to get updated counts for each recommended intervention for heart disease, as shown in Table 1.

**Table 1.** Heart disease PubMed recommendation table: counts for each recommended intervention

Heart disease intervention	Count
Aerobics	228
HIIT	103
Strength training	59

### Similarity search

We next determined whether the recommendations found in PubMed data appeared in UCI Health ED EHR data. We searched the summarized EHR data in two ways: one on the overall dataset and one filtered for heart disease based on a combination of ICD-10 codes in structured data and mentions of heart disease in clinician notes. As a reminder, the overall dataset had treatment plans that mentioned aerobic exercise or strength training, while the heart disease-specific treatment plans had mentions of heart disease and of aerobic exercise or strength training. When filtering for only ICD-10 codes for heart disease, only 82 records were retrieved, likely because an underlying condition is less likely to be why patients visit the ED; hence, these conditions are unlikely to be used for billing purposes. The list of ICD-10 codes used for filtering can be found in Table 2. That being said, heart disease as an underlying condition can cause symptoms that result in a patient visit, like chest pain or shortness of breath [32].

**Table 2.** Heart disease ICD-10 codes found in the EHR data

ICD-10 codes	Count
I25.10, I25.83, I25.89, I25.118, I25.5, I25.81, I25.2, I25.119, I25.709, I25.719, I25.810, I25.89, I25.9	82

The first round of similarity search provided a benchmark for how often exercise was included in medical records as an intervention in ED treatment plans; the results are shown in Table 3. The second round of similarity search focused specifically on heart disease and resulted in 432 records. No semantically similar treatment plans for aerobic exercise and strength training were found. To corroborate this, we manually searched the data as shown in Table 4. Exercise-themed recommendations appeared in less than 3% of heart disease-related medical records and only .034% of EHR treatment plans. After reviewing our findings with the UCI Health ED physicians, they noted that discussing diet and exercise with a patient they were admitting would be unlikely. They would be more likely to discuss these matters with a patient being discharged from the ED, though this may not be reflected in the EHR.

**Table 3.** Similarity search on overall EHR dataset

Intervention type	Example text	Distance	Count
AER: “practice more aerobic exercise”	“exercise through combination of aerobic/resistance/weight training”	119.05 – 210.37	472
AER: “practice more aerobic exercise like walking or jogging”	“aerobic activities. These are exercises, such asbrisk walking or water aerobics”	109.11 – 208.11	389
AER: “exercises like walking or jogging”	“exercise/movement: fast paced walking, swimming, running, biking/cycling, aerobic circuits”	144.78 – 253.14	124
STR: “increase muscle strength”	“PT services to increase strength and endurance, increase static and dynamic balance”	61.87 – 223.34	381
STR: “exercise like strength training”	“exercise; Patient education; Progressive resistive training; Strengthening exercises.”	109.17 – 210.78	195

**Table 4.** Manual search on heart disease EHR dataset

Intervention type	Treatment plan	Count
Aerobics	“if you joints are healthy, you do running, stairmaster, play sports” “150 minutes of moderate, high intensity aerobic exercise weekly” “walk 5 – 10 minutes twice a day”	7
Strength	“exercises; resistive band exercises; strengthening”	2

## Discussion

We demonstrated the potential utility of combining state-of-the-art searches of the medical literature with the analysis of natural language in the EHR to gauge the translation of clinical research knowledge into practice. The case of exercise and heart disease in the context of a busy ED demonstrated what appears to be a low level of translation, a finding consistent with our initial hypothesis. While the gap in translation of discovery to practice has been highlighted over the past several years, a major challenge in the field has been to develop metrics that can gauge improvement. The goal of this paper is to create a novel strategy to assess translation from bench to bedside. Without prior studies and prior metrics to compare, it is difficult to provide a quantitative score for how our metric performed. The hope is to use this method to create a metric to compare future levels of translation. Presented here is an approach to quantify clinical research translation by linking two of essential data sources in the clinical translation ecosystem, the biomedical research literature found in PubMed with patient data found in the electronic health record. This metric can then be used to 1) compare translation effectiveness between different diseases and conditions; 2) gauge the implementation of new diagnostic or therapeutic procedures; and 3) identify specific roadblocks in the translation process for individual diseases or health conditions.

The use of AI technology in this context might stimulate future applications in this field. Our text analytics approach introduced a self-supervised framework that is particularly suitable for highly specialized domains or situations with limited time or resources.

This approach enhances the effectiveness of the research and ensures easy reproducibility. The results of this research, in conjunction with discussions among physicians, clinical translational researchers, and basic scientists, lead us to propose the following ideas to bridge the gap between biomedical discovery (as represented in the literature) and clinical practice (as represented in the medical record). First, expand dissemination paradigms to websites, venues, podcasts, and listserv-based information opportunities. Second, use patient trust in primary care clinicians to spread research on exercise medicine, diagnostics, and therapeutic discoveries to the larger population. For instance, clinicians and researchers may develop pamphlets, blogs, short videos for clinician encounters in ED waiting areas. And third, ensure that exercise medicine knowledge contributes to mitigating health disparities and inequalities. Clinically relevant exercise science discoveries can be presented at staff/faculty meetings at Federally Qualified Health Centers/Family Health Centers. The impact of these strategies can then be tested using the bibliometric-medical record approach outlined in this publication.

Few efforts have been made to employ literature search technologies to gauge translational speed or effectiveness. The most relevant similar study we were able to identify was the novel work of Hutchins *et al*. [33] In building a machine learning system that detects whether a published article is likely to be cited in a future clinical trial or guideline. They found that unique knowledge flow trajectories could be associated with publications that either succeed or fail to influence clinical research downstream. The authors suggested that translational progress in medical research might actually be predicted by the publication community’s early reaction to a new publication. Our work extends this concept to a bibliometric and EHR-natural language strategy that gauges the translation of medical literature to actual clinical practice.

Our results in this use case suggest that physicians in the ED rarely mention exercise as a treatment plan in EHRs for patients with heart disease. For heart disease, aerobic and strength training exercises were included in only 0.34% of treatment plans, while across the entire dataset, exercise recommendations were included in treatment plans just 5% of the time. While these findings may reflect a disconnect between research and clinical practice, particularly in lifestyle interventions, other mechanisms are also possible.

The frontline ED clinicians who comprised our research group also highlighted additional factors that contribute to the apparent disconnect between research findings and clinical practice. ED physicians are under tremendous pressure to focus on the most seriously ill, which limits time for patients with less urgent cases. This can result in missed opportunities to provide patients with guidance that could lead to improved health outcomes and disease prevention. These issues are particularly significant post-COVID-19 pandemic, where patients now present with a range of health concerns that were not addressed during the peak of the COVID-19 outbreak.

The burden of using the EHR by frontline clinicians may have influenced our results. From the documented frustration of the not uncommon occurrence of transition to EHRs [34] to the unresolved feeling of “burn-out” experienced by many physicians [35], the EHR may not be the most accurate or dynamic reflection of the physician–patient interaction. Indeed, Hammond and colleagues recently argued that, “The Electronic Health Record has failed to meet its intended purpose,” as it was designed without input from patients and practitioners and for service functions rather than on managing patient data [36]. Consequently, information casually exchanged between patients and busy ED physicians may never be recorded in the EHR. As generative large language models are increasingly incorporated in the EHR, the need for caution surrounding “hallucinations,” ethical, and health equity concerns are growing [37]. The tools we present here may prove useful not only in quantifying translational effectiveness but also in gauging the quality of biomedical databases and the EHR itself.

### Limitations

Limitations of this study include the potential information loss resulting from summarizing the EHR treatment plans. To check for loss, we manually reviewed a sample of the summarized treatment plans and did not identify any major information losses, but further testing on potential loss could be a future step in this research. Summarized PubMed results sections are less of a concern as they are already condensed to fit into the abstract format, making it less likely to lose important information due to summarization.

Another potential limitation is the low results from the semantic similarity searches on the EHR dataset. Comparing the semantic similarity search with a manual search showed nine misses. A solution could be to switch the embedding or semantic search models to examine if one might perform better by more accurately catching these occurrences. Another solution could be to work with medical professionals to create more domain-specific search queries to identify interventions in treatment plans. Involving medical professionals could lead to more relevant search queries with medical verbiage or formatted in ways more likely to be seen in ED medical records. The margin of error is low, with less than 3% of abstracts containing these interventions after the manual search, so confirming with stakeholders what level of error they are comfortable with could be enough.

## Conclusions

Having access to publicly available clinical research and associated electronic health record data, including clinician notes and after-visit summaries, provided a unique opportunity to assess the effectiveness between clinical research and its adoption in medical practice. This self-supervised framework for direct query-based search can be used for a wide variety of clinical conditions and, if assessed over time, could measure the effectiveness of implementation strategies and clinical guidelines.
